# A novel epidemiological model to better understand and predict the observed seasonal spread of Pestivirus in Pyrenean chamois populations

**DOI:** 10.1186/s13567-015-0218-8

**Published:** 2015-07-24

**Authors:** Gaël Beaunée, Emmanuelle Gilot-Fromont, Mathieu Garel, Pauline Ezanno

**Affiliations:** INRA, Oniris, LUNAM Université, UMR1300 BioEpAR, CS40706 F-44307 Nantes, France; Université de Lyon, VetAgro Sup-Campus Vétérinaire de Lyon, Marcy l’Etoile, France; Université de Lyon, Université Lyon 1, UMR CNRS 5558 Laboratoire de Biométrie et Biologie Evolutive, Villeurbanne, France; Office National de la Chasse et de la Faune Sauvage, Centre National d’Études et de Recherche Appliquée Faune de Montagne, Gières, France

## Abstract

Seasonal variations in individual contacts give rise to a complex interplay between host demography and pathogen transmission. This is particularly true for wild populations, which highly depend on their natural habitat. These seasonal cycles induce variations in pathogen transmission. The seasonality of these biological processes should therefore be considered to better represent and predict pathogen spread. In this study, we sought to better understand how the seasonality of both the demography and social contacts of a mountain ungulate population impacts the spread of a pestivirus within, and the dynamics of, this population. We propose a mathematical model to represent this complex biological system. The pestivirus can be transmitted both horizontally through direct contact and vertically in utero. Vertical transmission leads to abortion or to the birth of persistently infected animals with a short life expectancy. Horizontal transmission involves a complex dynamics because of seasonal variations in contact among sexes and age classes. We performed a sensitivity analysis that identified transmission rates and disease-related mortality as key parameters. We then used data from a long-term demographic and epidemiological survey of the studied population to estimate these mostly unknown epidemiological parameters. Our model adequately represents the system dynamics, observations and model predictions showing similar seasonal patterns. We show that the virus has a significant impact on population dynamics, and that persistently infected animals play a major role in the epidemic dynamics. Modeling the seasonal dynamics allowed us to obtain realistic prediction and to identify key parameters of transmission.

**Electronic supplementary material:**

The online version of this article (doi:10.1186/s13567-015-0218-8) contains supplementary material, which is available to authorized users.

## Introduction

Pathogen spread in both human and animal populations is largely constrained by seasonal variations in individual contacts [1]. In animal populations, animal densities and population structure may vary with the availability of natural resources [2,3]. When resource becomes scarce, a cluster of individuals may appear (e.g., around waterholes [4] or on patches of snow-free vegetation). Conversely, sexual segregation, i.e., the separation of males and females by habitat, spatially or socially outside of the breeding season, is also a common phenomenon among a large range of animal species [5]. Such variations in grouping patterns impact the occurrence of both direct host-to-host contacts and indirect contacts through a shared contaminated environment or via a vector (e.g. [6,7]). In addition, most organisms live in a seasonal environment so that host demography is often seasonally determined [8]. Sexual contacts occurring only during a breeding season induce an annual pulse of births and thus a seasonal renewal in susceptible individuals. In addition, in host populations whose dynamics is driven by density-dependent processes, seasonal mechanisms, such as harvesting, could lead to compensatory mechanisms (e.g. higher birth rate) with consequences on pathogen spread [9]. The seasonality of the biological processes involved therefore should be considered to better represent and predict pathogen spread in a seasonal environment [1].

The genus Pestivirus, classified within the *Flaviviridae* family, comprises viruses that are major pathogens for both wild and domestic ungulates [10], and which can cross species barriers to infect a wide range of hosts [11,12]. In domestic species (and probably in wild species too), pestiviruses are a significant cause of reproductive failures such as abortion and stillbirths [13]. They may also have immunosuppressive effects, which increase the severity of other opportunistic infections [14]. Pestiviruses have a considerable impact on domestic and wild ungulate populations, and consequently represent a major economic challenge [11,15]. For example, classical swine fever occurs in domestic pigs and wild boars and causes major economic losses, particularly in countries with an industrialized pig production sector [12,16].

In Pyrenean chamois (*Rupicapra pyrenaica pyrenaica*) populations, severe outbreaks of disease associated with one Pestivirus, Border Disease Virus (BDV), have been reported since 2001 in Spain, Andorra and France [17]. This virus has caused mass mortality in some areas (notably in Spain [18]), and has become endemic in others. A decade after the first epidemic in Spain, some Pyrenean chamois populations quickly recovered after BDV transmission ceased, whereas in others continuous transmission was observed with possible detrimental effects on population dynamics [19]. To understand this complex dynamics, related to the interplay between host population dynamics and viral transmission, several key parameters are lacking, such as infection-related mortality. The impact of this pathogen on the population dynamics of wild ungulates remains still largely unknown and difficult to measure, but must be estimated and taken into account to improve the management of ungulate populations.

It has been shown that the spread of a Pestivirus depends on the season and the age structure of the population involved [17]. The internal structure of the population (seasonal breeding, heterogeneous contact structure) must therefore be considered in representations of virus spread. Modeling has been used for closely related systems, i.e., classical swine fever in wild boar [9] and pestiviruses in domestic ruminants [20]. However, these models do not simultaneously take into account seasonal variations in demography and heterogeneity of contact between individuals.

Our objective was to better understand how the seasonality of both demography and social contacts impacts the spread of one Pestivirus, BDV, in a mountain ungulate population and its population dynamics. We propose a deterministic compartmental mathematical model to represent this complex biological system. Modeling is a complementary tool to observational and experimental studies, especially for wildlife populations where individual monitoring of infection is lacking. Here, the model brings new elements to the field. Specifically, we evaluated whether including the seasonal host population dynamics and contact pattern that can be inferred from the knowledge of the species could result in the seasonal epidemiological variations that have been documented in the field [17]. We performed a sensitivity analysis of this model (i.e., we studied the effect of parameter variations on model outputs) to identify key parameters which are potential control points of the biological system and also sources of model uncertainty. Moreover, we produced first estimates of epidemiological parameters, namely transmission and infection-related mortality rates, and tested which were the most important ones for viral dynamics. For this, we used data from a long-term demographic and epidemiological survey of a Pyrenean chamois population at Orlu, France. All these elements participate in a better understanding of virus transmission and impact, a prerequisite for disease management. As far as we know, our study is the first to model the propagation dynamics of a Pestivirus in a Pyrenean chamois population.

## Materials and methods

### Pyrenean chamois population dynamics and BDV infection

The Pyrenean chamois has a highly seasonal reproductive cycle [21]. Births occur in May and June. The mating season lasts from November to early January, during which contact between males and females is the most frequent whereas sexual segregation is much more marked during the rest of the year [22].

In addition to seasonality, demographic parameters are affected by density-dependence. In wild ungulates, the mortality rate of juveniles and the fertility rate of subadults are the first parameters to be lowered at high density [23]. This is due to fertility being dependent on body weight, which is itself influenced by the population density and environmental conditions encountered the year of birth (e.g., in chamois [24]).

Like other pestiviruses, BDV has a limited survival in the environment [25]. Horizontal transmission mainly occurs through direct contact between susceptible and infected animals. Infection starts with a phase of transient viremia, with infected animals shedding low amounts of the virus before becoming immune. However, if infection occurs during the first half of pregnancy, this leads to abortion or vertical transmission [26]. In domestic sheep, vertically infected newborns are persistently infected (PI), and shed large amounts of virus during their entire life. Although PI animals have a high mortality, they constitute the largest source of the virus in a population, at least in domestic species [20]. PI females always give birth to PI newborns.

In the Pyrenean chamois, three experiments were conducted which confirmed some of the observations made in domestic species: the infection of a pregnant female confirmed the possibility of the birth of PI animals [27] and the infection of individuals carrying antibodies showed the existence of acquired protective immunity [28]. Viremia asted 34 and 51 days, respectively, in two experiments [28,29] but its maximal duration could not be measured due to experiment termination.

### Biological data

The data used to parameterize the model were obtained from surveys of a population located in the National Game and Wildlife Reserve of Orlu in the eastern French Pyrenees (42° 39.5' N, 1° 57.9' E). This population has been monitored intensively using the Capture-Mark-Recapture method since 1984 by the Office National de la Chasse et de la Faune Sauvage allowing detailed estimation of vital rates [30] (Table 1) and providing individual data on pestivirus infection [17]. This monitoring has been performed in accordance with the ethical conditions detailed in the specific accreditations delivered by the Préfecture de Paris (prefectorial decree n°2009-014) in agreement with the French environmental code (Art. R421-15 to 421–31 and R422-92 to 422-94-1). Variation in population size has been approximated since 1984 through yearly censuses in late spring as we were only interested in the global population trend, and as local CMR data would have most likely not provided much more reliable estimates at the population level.

**Table 1:** **Parameters of the model of pestivirus spread in a Pyrenean chamois population**

θ	Description (dimension)	Value	Ref.
Demographic parameters			
*η*_*Sa*_^*max*^	Fertility rate of sub-adult females, maximum (annual)	0.65	[21]
*η*_*A*_	Fertility rate of adult females (annual)	0.90	[21]
*η*_*SaP*_^*max*^	Fertility rate of sub-adult PI females, maximum (annual)	0.65	[21]
*η*_*AP*_	Fertility rate of adult PI females (annual)	0.90	[21]
*μ*_*Juv*_^*min*^	Probability of juvenile mortality, minimum (annual)	0.342	[21,31]
*μ*_*Juv*_^*max*^	Probability of juvenile mortality, maximum (annual)	0.85	^a^
*μ*_*Sa*_^*female*^	Probability of mortality of sub-adult females (annual)	0.1	^c^
*μ*_*Sa*_^*male*^	Probability of mortality of sub-adult males (annual)	0.171	^c^
*μ*_*A*_^*female*^	Probability of mortality of adult females (annual)	0.105	^c^
*μ*_*A*_^*male*^	Probability of mortality of adult males (annual)	0.143	^c^
*μ* ^*P*^	Probability of mortality of PI animals (annual)	0.75	[34]
*μ* ^*T*^	Probability of mortality related to a transient infection (over the duration of viremia)	0.196	^c^
*δ*	Sex ratio	0.5	^b^
*K*	Carrying capacity	3000	^a^
*d*	Strength of density dependence	0.8 × 10^−3^	^a^
Epidemiological parameters			
*1/α*	Duration of immunity by maternal antibodies (days)	60	^b^
*β*_*T*_	Horizontal transmission coefficient by a transiently infected animal (per day)	0.03	[34]
*β*_*P*_	Horizontal transmission coefficient by a PI animal (per day)	0.5	[34]
*1/γ*	Duration of viremia (days)	51	[29]
*1/ω*	Duration of immunity (years)	8	[28]
*ρ*	Probability of abortion	0.5	[26]
*ν*	Possibility of infection during the gestation period (boolean)	0/1	
*τ*	Indicator of rut (boolean)	0/1	

^a^Calibrated using field data.

^b^Experts knowledge.

^c^Unpublished data from the study site.

For the epidemiological survey, 535 individuals in total were captured (between April and July) or hunted (between August and December) each year between 1995 and 2010. For each individual, the age, sex and season of capture (spring for captures and autumn for hunting) were available. All individuals were tested for antibodies using the ELISA BVD/Mucosal Disease p80 kit (Institut Pourquier, Montpellier, France, see [17] for details). The presence of the virus in the population was detected since the epidemiological survey began in 1995, while a decline in population size was recorded, starting around the same year. However, the exact date of virus introduction is unknown. In such a context, understanding the determinants of disease transmission is a pre-requisite for the development of a management policy.

### The demographical-epidemiological model

Based on the available knowledge concerning the clinical and epidemiological aspects of BDV, we built a deterministic compartmental model describing BDV spread within a Pyrenean chamois population. The population was structured by age and sex. Three age classes were considered: juveniles ([0-1] year, i.e., kids), subadults ([1-2] years, i.e., yearlings) and adults (≥2 years). This was justified by the social structure and the observed reproduction pattern due to sexual maturity. As compared to typical age-classes commonly described for adults in ungulates (i.e., prime-aged adults (2–7 years old), old adults (8–12 years old) and senescent individuals (after 12 years old – [31]), we pooled adults (≥2 years) in a single age class for model simplicity.

In the population studied, some females were able to breed at 1.5 years old with a calving at 2 years of age (subadults). However, final maturity was most often reached the following year [21]. Due to seasonal births (May-June), the transitions between age classes were considered to occur on July 1^st^. We represented the life cycle of a Pyrenean chamois as follows: an average of 1 year and 1 month as a juvenile (from birth to the end of June the following year), 1 year as a subadult, and the rest of the life cycle as an adult. The juvenile class was divided into two subclasses: newborns (in May and June of the year of birth) and young (from July 1^st^ to June 30), in order to avoid overlapping between cohorts.

We used two versions of the model: one accounting for the social behavior and the dynamics of the group structure as heterogeneous contacts may influence the infection spread (see Additional file 1), one assuming homogeneous contacts all year long. In the heterogeneous case, all individuals were able to meet each other during the mating season (from November to early January). After this period, groups were formed and contacts were assumed to be heterogeneous: adult females formed one group with juveniles and subadult females while adult males were considered to form a separate group [23,32,33]. Subadult males were assumed to stay with either the group of adult males or that of females and juveniles, thus having contact with both.

**Figure 1:**
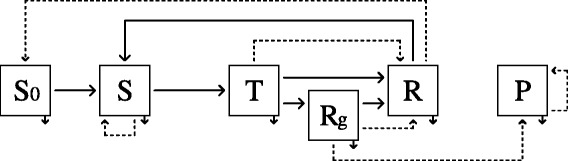
**Conceptual model of pestivirus spread.** Squares: health states, solid arrows: transitions between health states, dashed arrows: reproduction (production of newborns), *S*_*0*_: protected by maternal immunity, *S*: susceptible, *T*: transiently infected, *Rg*: resistant with a possibility of pregnancy with a risk of vertical transmission, *R*: resistant without a possibility of pregnancy with a risk of vertical transmission, *P*: persistently infected. This scheme is broken down for each age class and sex with some variations: compartment *S*_*0*_ is present only for newborns and young animals; compartment *Rg* is present only for sub-adult and adult females.

We considered in the model five health states (Figure 1): *S*_*0*_, kids protected by maternal antibodies, *S*, susceptible to infection, *T*, transiently infected, *R*, resistant (protected by immunity developed after infection), and *P*, persistently infected. Hence, by age and sex, each health state corresponded to a model compartment, except for subadult and adult females, for which the *R* compartment was divided into two compartments: *Rg* and *R* (see the extended representation of the conceptual model in Additional file 2). A female infected during her pregnancy went into the *Rg* (*R* gestation) compartment at recovery and remained there until the end of the birth period. Otherwise, infected animals went into the *R* compartment at recovery.

Only the subadult and adult females were able to breed and give birth to newborns whose health state was determined by the state of the mother. Vertical transmission was possible during pregnancy, depending on the date of maternal infection. A female infected during the first half of pregnancy was assumed to abort (with probability *ρ*) or to give birth to a *P* newborn (with probability 1-*ρ*). When a female was infected during the second half of pregnancy, the fetus was immuno-competent and therefore able to fight infection. The pregnancy thus resulted in a resistant newborn (*R*). All *P* dams gave birth to *P* newborns, *S* females give birth to *S* newborns while *R* females produce *S*_*0*_ newborns. In addition, as orphans had a very low chance of survival, at each time step, the mortality events occurred before birth (to consider only individuals still alive).

The ordinary differential equations (ODE) system describing the rate of change in each health state is given in Additional file 3. The transitions between age groups were not included in these equations because they were considered as discrete time events and took place each year just after the birth period (on July 1^st^). A semi-implicit Euler method was used for the discretization of the ODE in time. Epidemiological and demographic processes were considered with a daily time step.

### Parameter estimation and model analysis

The demographic parameters (Table 1) were calibrated by integrating knowledge from unpublished and published data on the focal population and by using experts’ knowledge. The mortality rate of juveniles and the fertility rate of subadult females are known to be strongly influenced by density-dependence processes in ungulate species [23]. We therefore used a sigmoid function to continuously represent variations in these rates using explicit variables (*d*: strength of density dependence, *K*: carrying capacity, *N*: total population size), (equations are given in Additional file 3).

Several epidemiological parameters (Table 1) were calibrated by integrating knowledge available on Pyrenean chamois populations. When parameters were unavailable for wild populations of ungulates, we used data from experiments on domestic species (mainly sheep).

Parameters that could not be obtained from Pyrenean chamois were disease-related mortality of *P* animals and transmission coefficients by both *P* and *T* individuals. For the first simulations, the probability of disease-related mortality for transiently-infected individuals was calculated using data from Pioz et al. (unpublished data). The survival of *P* animals was based on a cattle study [34], and corresponds to a half-life of 6 months. For the transmission coefficients, the only information available concerned cattle, and we chose as priors the parameters estimated for Bovine Viral Diarrhea Virus (BVDV) in this species [34].

As initial conditions, the composition of the population (age and sex) was determined on the basis of the stable age structure predicted by simulations without introduction of the pathogen, and population size was based on the yearly censuses. In the following, population size estimates provided by our model are thus estimates of the yearly censuses. The virus introduction was simulated by introducing a persistently infected newborn kid in the middle of the birth period. Such an introduction corresponds to the birth of a PI newborn whose mother would have been infected during the first part of gestation (e.g. through contact with a neighboring chamois population or domestic livestock). Preliminary simulations had shown that introducing a single transiently infected chamois does not lead to virus spread in the population and was therefore not further explored.

To determine the parameters that contributed to variations in model outputs, a sensitivity analysis was performed using the Fourier Amplitude Sensitivity Test (FAST) [35]. This analysis assesses the extent to which model predictions are influenced by variations in model parameters. Two main outputs of the model were discussed: the seroprevalence during the endemic period and the decrease in population size subsequent to the virus introduction. The decrease in population size was assessed by the final population size assuming no virus spread minus the final population size assuming virus spread. The main effects and interactions between parameters were quantified by allowing all parameters to vary simultaneously. Each parameter varied between −25% and +25% of its nominal value, which generated 10 000 scenarios for each parameter. As other parameters also varied, a total of 190 000 scenarios were generated. The sex ratio at birth was not taken into account in the analysis. For each output, a linear regression model was fitted with all of the principal effects and the first-order interactions [35]. The overall contribution of factor *i* to variations in output *y* was the ratio of the sum of square of the linear regression model for output *y* related to the principal effect for factor *i* plus half the sum of squares related to interactions involving factor *i* over the total sum of squares of the linear model. The sum of the contributions of all the factors for a given output was equal to the coefficient of determination of the regression model R^2^. We considered a parameter as a key parameter if it contributed to at least 10% of the variance of one of the model outputs.

Estimates of the coefficients of horizontal transmission and the probability of disease-related mortality were then refined using available data on seroprevalence (see Additional file 4) and a demographic survey involving Approximate Bayesian Computation (ABC). The ABC method consists of studying the similarity between observed and simulated data. The most probable set of parameters is obtained when the simulated data are the closest to the observed data. The algorithm used was the ABC rejection sampler [36,37]. A major advantage of the ABC method is that it does not require the specification of a likelihood function [38], which would have been impossible in such a complex model. The range of parameter values used for the estimation of the transmission coefficients was chosen relative to data from cattle [39]. Since the proximity between individuals and contact rates are high in cattle, we expected transmission coefficients to be lower in a wild-living population compared to domestic species. The ranges used for the estimation were thus [0.05, 0.6] for β_P_ and [0.001, 0.04] for β_T_. Wide ranges were used for the probability of disease-related mortality during viremia μ_T_): [0.01, 0.99]. The main source of bias that may occur with the use of ABC comes from the choice of the summary statistics and the method of comparison between observed and simulated data. The use of two types of data with different orders of magnitude led us to use a distance of χ^2^. It has the advantage of being weighted and therefore makes the distances comparable. The summary statistics were chosen to maximize information and accuracy. Other techniques exist [20,40] that allow more accuracy in estimating parameters, but they require the chronological monitoring of the same animals, which was not feasible here.

The scenarios (*n* = 100 000) were defined by random sampling in uniform distributions over these ranges. Moreover, as the exact year of virus introduction was not known, several options were tested from 1990 to 1993. To assess the importance of PI individuals in the epidemiological dynamics, we performed an additional estimation considering a model without persistently infected animals. In this case, the range used for β_T_ was larger [0.001, 0.6], β_P_ value was 0, and the risk of abortion when infection occurs during the first half of pregnancy was 100%. Lastly, to evaluate the impact of assuming homogeneous or heterogeneous contacts, we performed an additional estimation considering homogeneous contacts all year long.

We used the chi-square distance to compare the observed and simulated data. Two sets of data were used: seroprevalence over the last eleven years, which corresponds to an endemic period, considering only animals tested for antibodies and viral antigen, aggregated by age classes and sampling seasons, and the estimates of population size over time. We selected the simulations producing the lowest distance between observed and simulated data with different rejection thresholds (1, 2, 3, 4, 5, 10, 20, 30, 40, 50% of the simulations).

## Results

### Preliminary model predictions and analysis

**Figure 2:**
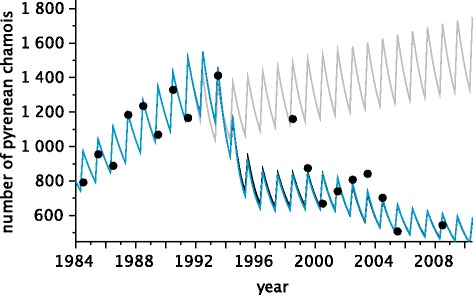
**Yearly censuses over time.** Circles: observed data. Curves: model predictions (in grey: simulations with parameter values before estimation; in black: simulations with parameter values estimated by ABC; in blue: simulations with parameter values estimated by ABC and assuming homogeneous contacts all year long).

**Figure 3:**
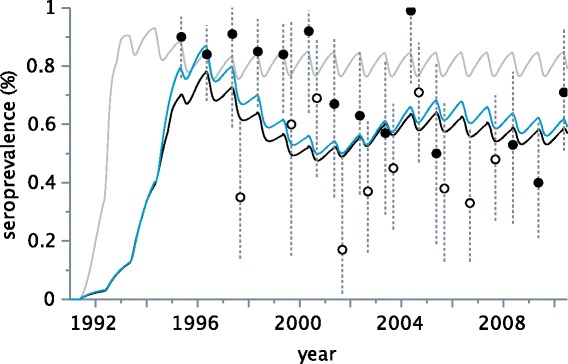
**Seroprevalence over time.** Circles: observed data (in black: spring; in white: autumn, dotted lines correspond to confidence bound intervals). Curves: model predictions (in grey: simulations with parameter values before estimation; in black: simulations with parameter values estimated by ABC; in blue: simulations with parameter values estimated by ABC and assuming homogeneous contacts all year long).

Preliminary simulations (Figures 2 and 3), which were based on the parameter values derived from the literature and experts’ knowledge, could not reproduce the demographic trend in the population of Orlu. In particular, simulations using a priori parameter values did not track the changes in population size after virus introduction. The variations in the endemic seroprevalence had the same pattern of alternating seasons between simulations and observations, with high values in the spring and low values in the autumn, but with weaker amplitudes.

**Figure 4:**
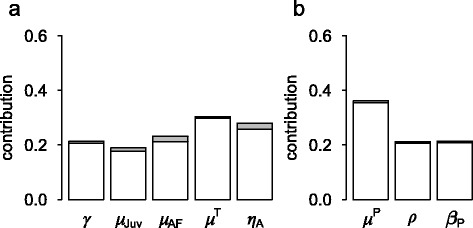
**Sensitivity analysis of two model outputs.** **A** Decrease in population size subsequent to the virus introduction, assessed by the final population size assuming no virus spread minus the final population size assuming virus spread, **B** seroprevalence during the endemic period. In white: the main effects; in grey: the interactions. We presented here only parameters contributing to at least 10% of the variance of one of the model outputs, considered as the most important model parameters.

The sensitivity analysis (Figure 4) indicated that parameters related to persistently infected animals were key parameters affecting the endemic seroprevalence. These parameters were the mortality of PI animals (μ^P^), the risk of abortion (ρ), and the coefficient of horizontal transmission by contact with a PI animal (β^P^), the latter being mostly unknown for Pyrenean chamois.

Five key parameters influenced the host population dynamics. Juvenile mortality (μ_Juv_), adult female mortality (μ_A_) and adult female fecundity (η_A_) have been calibrated on data, and therefore the uncertainty related to their value is moderate. In contrast, the disease-related mortality for transiently infected animals and the duration of viremia are directly related to the epidemics, and the disease-related mortality rate is by far the most uncertain parameter.

### Parameter estimation by ABC

**Figure 5:**
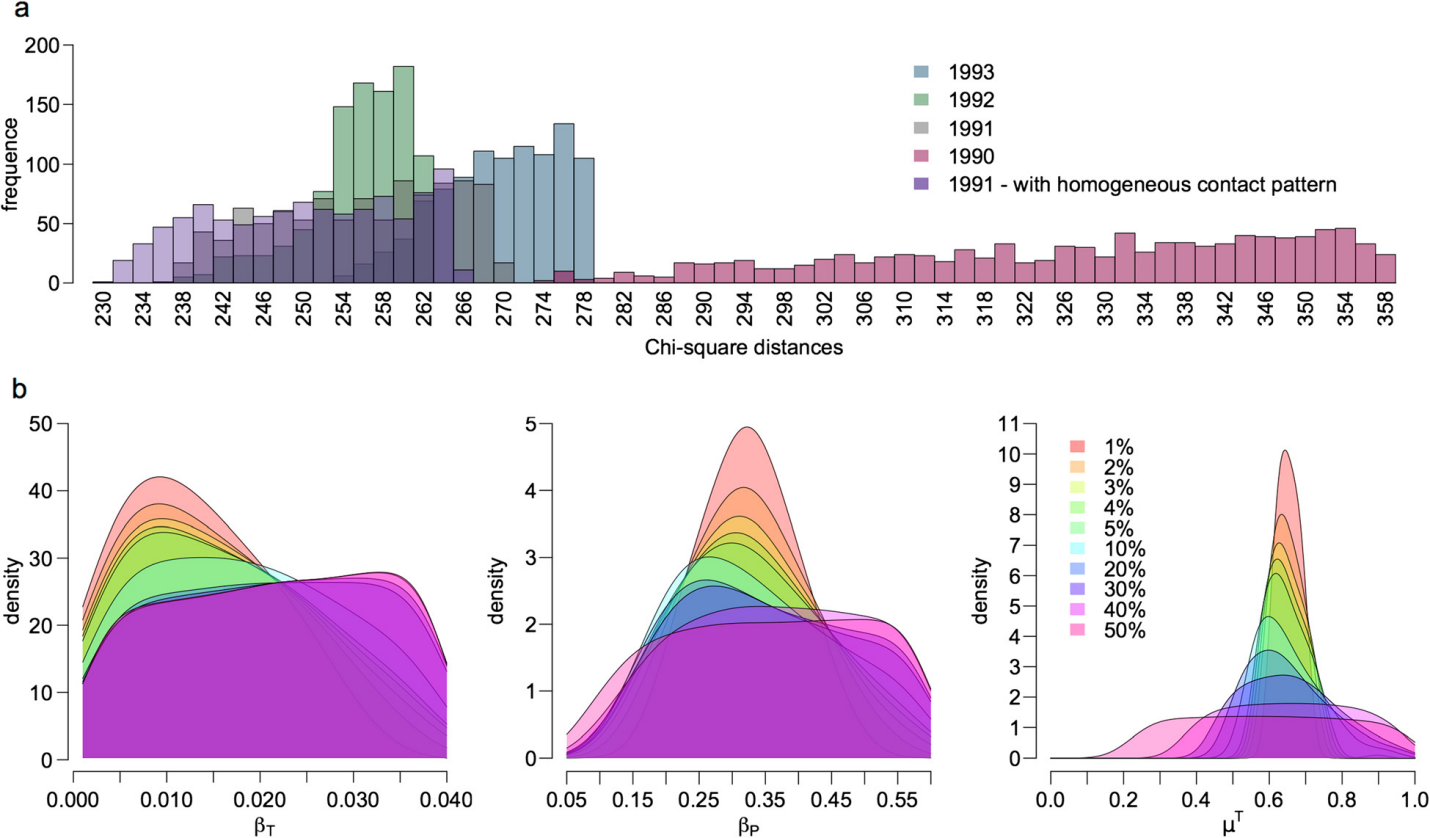
**Parameter estimation by ABC.** **A** Distributions of chi-square distances between simulated and observed data, using the 1% rejection threshold, depending on the year of virus introduction (red: 1990, grey: 1991, green: 1992, blue: 1993, purple: 1991 but assuming homogeneous contacts all year long). **B** Distributions of probability density for different rejection thresholds for a virus introduction in 1991, assuming heterogeneous contacts, and when PI are considered in the model: β_T_ the coefficient of horizontal transmission from transiently infected animals, β_P_ the coefficient of horizontal transmission from persistently infected animals, and μ^T^ the disease-related mortality of transiently infected animals.

For year of virus introduction, 1990 gave the highest distances between simulations and observations. For the three other introduction dates tested, differences were smaller (Figure 5A). Years 1991 and 1992 gave the best results, year 1991 giving predictions closest to observations, assuming or not a seasonal contact pattern.

For introduction years 1991 to 1993, the estimated parameter values are given in Table 2. The distributions of the probability density of the parameter values for different thresholds for year 1991 are shown in Figure 5B.

**Table 2:** **Estimation of transmission rates by transiently (**β_T_ and persistently infected (β_P_) animals, and disease-related mortality rate (μ^T^) using Approximate Bayesian Computation (ABC)

	Parameters		
Year of virus introduction	*β*_*T*_	*β*_*P*_	*μ* ^*T*^
1991	0.009 [0.002; 0.028]	0.32 [0.20; 0.46]	0.64 [0.60; 0.70]
1992	0.032 [0.003; 0.039]	0.32 [0.20; 0.43]	0.66 [0.60; 0.70]
1993	0.010 [0.002; 0.035]	0.37 [0.24; 0.51]	0.69 [0.67; 0.75]
1991 – homogeneous contact	0.009 [0.002; 0.028]	0.35 [0.23; 0.50]	0.64 [0.60; 0.70]

Three years of virus introduction were tested. For the most probable year of introduction, estimation was also carried out with the model assuming homogeneous contacts all year long. Median and 95% confidence intervals (in brackets) are provided. Units are the same as in Table 1.

Estimations with the model without PI produced distances between observations and simulations that were much higher than estimations with the model with PI (see Additional file 5). This result illustrates that PI individuals, although rarely detected in field studies, are probably determinant in disease dynamics, and their absence is unlikely.

### Predictions with the estimated parameter values

Simulations with the new parameter values (Figures 2 and 3) produced predictions quantitatively and qualitatively more in accordance with observed data than the preliminary simulations, irrespective to the contact pattern.

**Figure 6:**
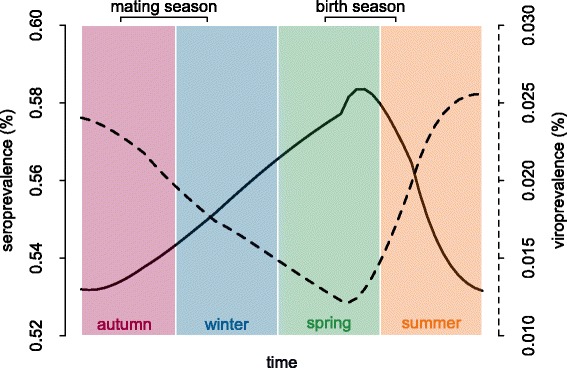
**Predicted seasonal pattern of seroprevalence and viroprevalence.** Plain line: seroprevalence; dashed line: viroprevalence.

The headcount over time predicted by the model was in good agreement with observed data, the decrease in population size after virus introduction being reproduced by the model. The seasonal pattern for seroprevalence was still present (Figure 6). When we observed seasonal variations more closely over the course of one year, after the birthing period there was a decrease in seroprevalence for about three months followed by an increase until the next period of births. The values and dynamics obtained, while a little higher, were nearer to the observations than those of the preliminary simulations. There was a sharp rise in seroprevalence followed by a decline and stabilization that correspond to the inter-annual variations observed however with lower amplitude.

## Discussion

This work was aimed at identifying the key determinants of the spread of BDV in populations of the Pyrenean chamois, with a particular focus on seasonality and on the role of PI individuals.

We first showed that accounting for seasonality in demographic processes and contacts allows model predictions to qualitatively match the seasonal variations of viroprevalence and seroprevalence as observed in the Orlu population [17]. Seasonality in pestivirus infection (Figure 6) thus probably results from an interaction between demographic and social patterns, with the following hypothetical scenario. At the end of spring, the birth of PI and susceptible kids allows transmission to start among juveniles. However, as a large proportion of adult females is immune, most juveniles carry maternal antibodies that protect them until the end of summer. A second arrival of susceptible juveniles thus occurs progressively in the summer and autumn, when maternal immunity fades out. Viral transmission is thus enhanced, first within groups of juveniles and females, and then between males and females during the rutting season [41]. Consequently, most infections occur in the autumn, which gives rise to the peak of viroprevalence observed at that time [17]. More precisely, the low seroprevalence observed in the autumn suggests that many transmissions probably occur relatively late in the season (Figure 3, white dots) and thus infections may continue in the winter. These infections acquired at the end of autumn probably terminate during the winter by the death or immunization of infected individuals, which is in line with the discovery of most carcasses between January and June [42]. Viroprevalence is thus at its lowest in the spring, while seroprevalence is the highest [17]. Due to strong herd immunity and low viroprevalence, horizontal transmission may cease in the spring, and start again only after the birth of PI kids.

While the model qualitatively predicted the observed seasonal pattern, the magnitude of predicted variations was lower than observed. The model also failed to reproduce the high level of inter-annual variability [17]. This discrepancy may be related to other causes of temporal variability that were not considered here, such as seasonal changes in immune defenses or the variations of demographic and social patterns that result from environmental stochasticity [1]. Small sample sizes also may contribute to explain the high variability of the observed values.

Second, the model revealed the importance of persistently infected animals in the spread of BDV in a Pyrenean chamois population, since the scenario with PI animals produced a better representation of epidemic dynamics than the scenario without such animals. Variables related to PI animals were also identified as key parameters affecting model predictions. In wild populations affected by BDV, the existence of PI animals is suspected but not proven, possibly due to their rarity and short lifespan. To our knowledge, it has not yet been possible to demonstrate persistent infection in captured animals, although experimental infections showed that vertical transmission and the birth of PI animals are possible [27,29]. In livestock, if most PI animals die quickly, some may survive for years and even reproduce. In chamois, the survival of PI individuals is unknown. Hence, we assumed that PI females may also survive and produce a kid, although this would constitute an exceptional event. To check whether our model allows for such events, we obtained the number of PI kids born to PI mothers from the simulations. This number was very low (~ one birth of PI kid to PI mother over 30 years of viral presence), which confirms that nearly all PI die before 2 years of age. Nevertheless, the survival of PI individuals across several seasons is a key mechanism of virus maintenance, as confirmed by the fact that the introduction of a single transitory infected chamois does not lead to virus spread in the population (result not shown). Our findings suggest that, although not easily observed, these individuals play a key role in maintaining infection. The role of PI has been demonstrated in other species, including in wild-living and for other pestiviruses [39]. Consequently, removing PI animals from the population as soon as they are detected has been identified as a major management tool in domestic species [11,43].

The model also brought new insight into the epidemics in Orlu and its impact on the host population. First, working under the assumption that the observed decrease in population size was largely due to the spread of the pestivirus, the test of different years indicated that the first case of PI was born in 1991, two years before the fall in population size, which means that the mother was likely infected in the autumn 1990. This delay between virus introduction and the decline of the host population, here between 1993 and 1996, is consistent with the retrospective analysis performed in Spain, which showed that the pestivirus was present in the chamois populations at least since 1990, long before the first observed major epidemic [44]. After virus introduction, our predictions showed an epidemic, with a sudden drop in population size, followed by an endemic situation accompanied by a continuous decrease in host populations. This continuous transmission after the first epidemic associated to a long-term decline of host population was consistent with the observations made in the Val d’Aran and Pallars Sobirà areas in Spain. However, other areas such as Cerdanya, Alt Urgell, Bergueda and Solsonès showed that populations recovering after virus transmission was possibly interrupted by high host mortality [19]. A deterministic approach is not appropriate to test this hypothesis, but a stochastic version of our model should help to firmly conclude on the existence of such a scenario.

Several epidemiological parameters had not been estimated before this study. The coefficients of horizontal transmission by PI and transiently infected animals and the probability of disease-related mortality for transiently infected animals were chosen to be estimated because they appeared to be among the most important parameters in the epidemic dynamics and also had the highest uncertainty. The use of an ABC approach allowed us to obtain parameter values consistent with existing knowledge concerning the pestivirus and the Pyrenean chamois. We estimated that 64% of the transiently infected animals will die over the course of the infection, a much higher value than initially evaluated (Table 1), but consistent with mass mortalities observed during several epidemics [18]. The determined values were estimated with variable accuracy, with the μ^T^ value estimated with a relatively high precision while the β_T_ and β_P_ values were estimated with a lower one. In comparison with the preliminary simulations, the estimated parameter values used allowed us to obtain a demographic trend that was much more consistent with the data, although the decline in the population size was not exactly the same. However, the headcount used was derived from ground counts, which are known to represent a variable under-estimation of the proportion of the true population size [45]. This means that trends in population sizes more than absolute values should be considered here in the biological interpretation. Our estimates of epidemiological parameters could now be used to represent the virus dynamics in other populations, and test whether the characteristics of each population and/or rare events represented in a stochastic framework may explain the emergence of several epidemiological scenarios according to the population considered [19].

In conclusion, this model allowed us to represent the spread of a pestivirus in a Pyrenean chamois population and to highlight the effect of seasonal demography and contacts on prevalence. Our results confirm that the virus has a significant impact on population dynamics, which reinforces the need to identify appropriate management actions, and that persistently infected animals play a major role in epidemic dynamics. Removing PI individuals when detected, a major tool of pestivirus management in domestic populations, is not feasible here, as these animals are not easily detected. Moreover, hunting may interfere with disease spread and impact, as demonstrated in wild boar populations [9]. Further modeling is required to better understand the impact of strategies such as non-selective harvesting, test-and-cull or vaccination, and their interaction with hunting, and to identify effective strategies to control the spread and impact of pestiviruses in natural populations.

## Additional files

Additional file 1:
**Contact structure.** This file contains information about the social behavior and the dynamics of the group structure, and a figure showing the contact structure, based on sex, age and season. Additional file 2:
**Conceptual model of pestivirus spread.** Figure showing the detailed conceptual model of pestivirus spread. Additional file 3:
**Associated equations of the model.** This file contains the ordinary differential equation system associated to the model. Additional file 4:
**Data used for estimation by ABC.** Tables containing the data used for the parameter estimations by the ABC method. Additional file 5:
**Parameters estimation by ABC.** Figure showing the distributions of distances between simulated and observed data.
