# Supplementary material for: A novel epidemiological model to better understand and predict the observed seasonal spread of Pestivirus in Pyrenean chamois populations
Source: Vet Res. 2015 Jul 24;46(1):86. doi: 10.1186/s13567-015-0218-8 (PMC4513621; doi:10.1186/s13567-015-0218-8)
Supplement: Additional file 1: — Contact structure. This file contains information about the social behavior and the dynamics of the group structure, and a figure showing the contact structure, based on sex, age and season. [file 13567_2015_218_MOESM1_ESM.pdf]

Additional file 1 - Contact structure

The social behaviour and the dynamics of the group structure were accounted for to model the infection spread. During the mating season (a), from mid November to early January, all individuals were able to meet each other, τ (rut indicator) is 0 during this season and otherwise 1. After this period, groups were formed and contacts were assumed to be heterogeneous (b) : adult females formed one group with juveniles and subadult females while adult males were considered to form a separate group. Subadult males were assumed to move between the group of adult males and that of females and juveniles, thus having contacts with both.

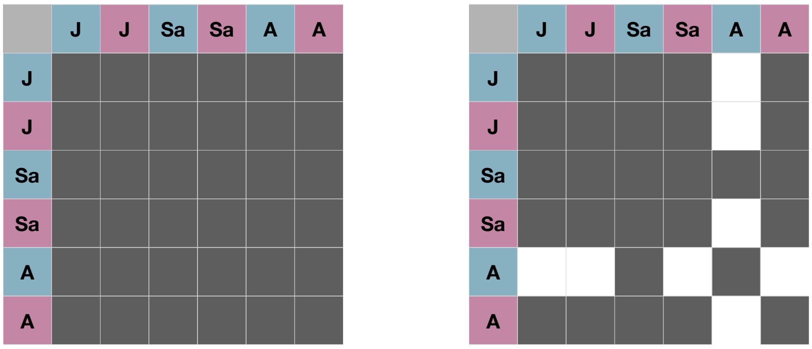

(a) (b)

Matrix contacts based on sex, age and season : during mating season (a) and out of mating season (b) ; Legend - black : possible contacts, white : no contact, J : juveniles, Sa : subadult, A : adult, blue : male, red female.
