# Supplementary material for: A novel epidemiological model to better understand and predict the observed seasonal spread of Pestivirus in Pyrenean chamois populations
Source: Vet Res. 2015 Jul 24;46(1):86. doi: 10.1186/s13567-015-0218-8 (PMC4513621; doi:10.1186/s13567-015-0218-8)
Supplement: Additional file 2: — Conceptual model of pestivirus spread. Figure showing the detailed conceptual model of pestivirus spread. [file 13567_2015_218_MOESM2_ESM.pdf]

Additional file 2 - Conceptual model of pestivirus spread

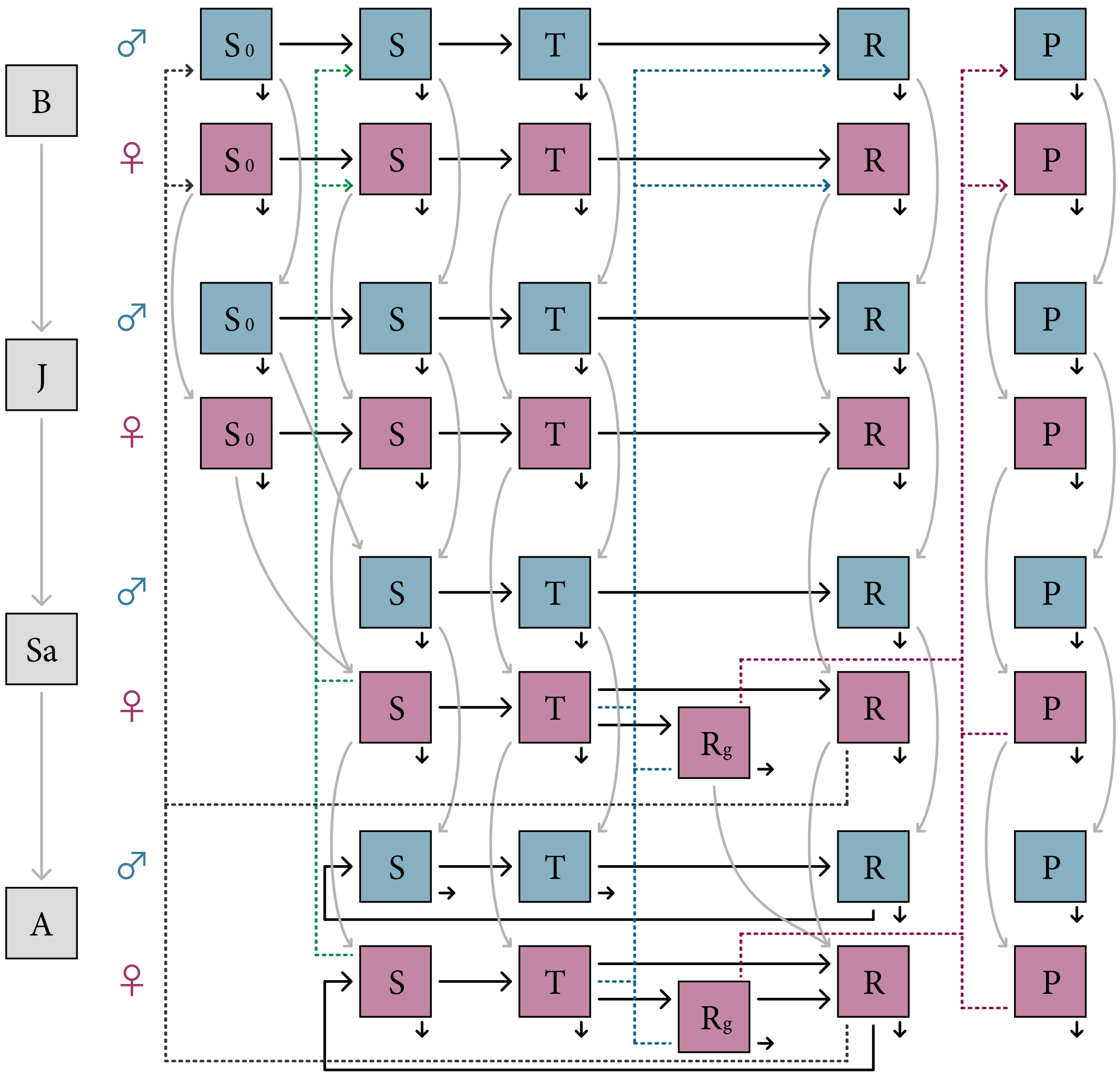

Conceptual model of pestivirus spread. Squares : health states, solid black arrows : transitions between health states, solid gray arrows : transitions between age classes, dashed arrows : reproduction (production of newborns), S_0 : protected by maternal immunity, S : susceptible, T : transiently infected, Rg : resistant with a possibility of pregnancy with a risk of vertical transmission, R : resistant without a possibility of pregnancy with a risk of vertical transmission, P : persistently infected.
