# Supplementary material for: A novel epidemiological model to better understand and predict the observed seasonal spread of Pestivirus in Pyrenean chamois populations
Source: Vet Res. 2015 Jul 24;46(1):86. doi: 10.1186/s13567-015-0218-8 (PMC4513621; doi:10.1186/s13567-015-0218-8)
Supplement: Additional file 3: — Associated equations of the model. This file contains the ordinary differential equation system associated to the model. [file 13567_2015_218_MOESM3_ESM.pdf]

Additional file 3 - Associated equations of the model

The ODE system is given below. The notation $\dot{x}$ denotes the derivative with respect to time such that $\dot{x} = \frac{dx}{dt}$. The proportion of females at birth is δ and the proportion of males is $(1-\delta)$. Functions of horizontal transmission (f_i) are defined in the following equations. The meaning of the parameters is given in Tables 1 and 2 of the article.

Newborn ♀

(1a) $\dot{S^0_{B♀}} = -(\mu_{Juv}(t)+\alpha).S^0_{B♀} + \delta.\left(\eta_{Sa}(t).R_{Sa♀} + \eta_A.R_{A♀}\right)$

(1b) $\dot{S_{B♀}} = -(\mu_{Juv}(t)+f_1).S_{B♀} + \alpha.S^0_{B♀} + \delta.\left(\eta_{Sa}(t).S_{Sa♀} + \eta_A.S_{A♀}\right)$

(1c) $\dot{T_{B♀}} = -(\mu_{Juv}(t)+\mu^T+\gamma).T_{B♀} + f_1.S_{B♀}$

(1d) $\dot{R_{B♀}} = -\mu_{Juv}(t).R_{B♀} + \gamma.T_{B♀} + \delta.\left(\eta_{Sa}(t).T_{Sa♀} + \eta_A.T_{A♀} + \eta_{Sa}(t).(1-p_{Sa♀}).R^g_{Sa♀} + \eta_A.(1-p_{A♀}).R^g_{A♀}\right)$

(1e) $\dot{P_{B♀}} = -(\mu_{Juv}(t)+\mu^P).P_{B♀} + \delta.(1-\rho).\left(\eta_{SaP}(t).P_{Sa♀} + \eta_{AP}.P_{A♀} + \eta_{Sa}(t).p_{Sa♀}.R^g_{Sa♀} + \eta_A.p_{A♀}.R^g_{A♀}\right)$

Newborn ♂

(2a) $\dot{S^0_{B♂}} = -(\mu_{Juv}(t)+\alpha).S^0_{B♂} + (1-\delta).\left(\eta_{Sa}(t).R_{Sa♀} + \eta_A.R_{A♀}\right)$

(2b) $\dot{S_{B♂}} = -(\mu_{Juv}(t)+f_1).S_{B♂} + \alpha.S^0_{B♂} + (1-\delta).\left(\eta_{Sa}(t).S_{Sa♂} + \eta_A.S_{A♀}\right)$

(2c) $\dot{T_{B♂}} = -(\mu_{Juv}(t)+\mu^T+\gamma).T_{B♂} + f_1.S_{B♂}$

(2d) $\dot{R_{B♂}} = -\mu_{Juv}(t).R_{B♂} + \gamma.T_{B♂} + (1-\delta).\left(\eta_{Sa}(t).T_{Sa♀} + \eta_A.T_{A♀} + \eta_{Sa}(t).(1-p_{Sa♀}).R^g_{Sa♀} + \eta_A.(1-p_{A♀}).R^g_{A♀}\right)$

(2e) $\dot{P_{B♂}} = -(\mu_{Juv}(t)+\mu^P).P_{B♂} + (1-\delta).(1-\rho).\left(\eta_{SaP}(t).P_{Sa♀} + \eta_{AP}.P_{A♀} + \eta_{Sa}(t).p_{Sa♀}.R^g_{Sa♀} + \eta_A.p_{A♀}.R^g_{A♀}\right)$

Young ♀

(3a) $\dot{S^0_{Y♀}} = -(\mu_{Juv}(t)+\alpha).S^0_{Y♀}$

(3b) $\dot{S_{Y♀}} = -(\mu_{Juv}(t)+f_1).S_{Y♀} + \alpha.S^0_{Y♀}$

(3c) $\dot{T_{Y♀}} = -(\mu_{Juv}(t)+\mu^T+\gamma).T_{Y♀} + f_1.S_{Y♀}$

(3d) $\dot{R_{Y♀}} = -\mu_{Juv}(t).R_{Y♀} + \gamma.T_{Y♀}$

(3e) $\dot{P_{Y♀}} = -(\mu_{Juv}(t)+\mu^P).P_{Y♀}$

Young ♂

(4a) $\dot{S^0_{Y♂}} = -(\mu_{Juv}(t)+\alpha).S^0_{Y♂}$

(4b) $\dot{S_{Y♂}} = -(\mu_{Juv}(t)+f_1).S_{Y♂} + \alpha.S^0_{Y♂}$

(4c) $\dot{T_{Y♂}} = -(\mu_{Juv}(t)+\mu^T+\gamma).T_{Y♂} + f_1.S_{Y♂}$

(4d) $\dot{R_{Y♂}} = -\mu_{Juv}(t).R_{Y♂} + \gamma.T_{Y♂}$

(4e) $\dot{P_{Y♂}} = -(\mu_{Juv}(t)+\mu^P).P_{Y♂}$

Sub-adult ♀

(5a) $\dot{S_{Sa♀}} = -(\mu_{Sa♀} + f_1).S_{Sa♀}$

(5b) $\dot{T_{Sa♀}} = -(\mu_{Sa♀} + \mu^T + \gamma).T_{Sa♀} + f_1.S_{Sa♀}$

(5c) $\dot{R^g_{Sa♀}} = -\mu_{Sa♀}.R^g_{Sa♀} + \nu(t).\gamma.T_{Sa♀}$

(5d) $\dot{R_{Sa♀}} = -\mu_{Sa♀}.R_{Sa♀} + (1-\nu(t)).\gamma.T_{Sa♀}$

(5e) $\dot{P_{Sa♀}} = -(\mu_{Sa♀} + \mu^P).P_{Sa♀}$

Sub-adult ♂

(6a) $\dot{S_{Sa♂}} = -(\mu_{Sa♂} + f_2).S_{Sa♂}$

(6b) $\dot{T_{Sa♂}} = -(\mu_{Sa♂} + \mu^T + \gamma).T_{Sa♂} + f_2.S_{Sa♂}$

(6c) $\dot{R_{Sa♂}} = -\mu_{Sa♂}.R_{Sa♂} + \gamma.T_{Sa♂}$

(6d) $\dot{P_{Sa♂}} = -(\mu_{Sa♂} + \mu^P).P_{Sa♂}$

Adult ♀

(7a) $\dot{S_{A♀}} = -(\mu_{A♀} + f_1).S_{A♀} + \omega.R_{A♀}$

(7b) $\dot{T_{A♀}} = -(\mu_{A♀} + \mu^T + \gamma).T_{A♀} + f_1.S_{A♀}$

(7c) $\dot{R^g_{A♀}} = -\mu_{A♀}.R^g_{A♀} + \nu(t).\gamma.T_{A♀}$

(7d) $\dot{R_{A♀}} = -(\mu_{A♀} + \omega).R_{A♀} + (1-\nu(t)).\gamma.T_{A♀}$

(7e) $\dot{P_{A♀}} = -(\mu_{A♀} + \mu^P).P_{A♀}$

Adult ♂

(8a) $\dot{S_{A♂}} = -(\mu_{A♂} + f_3).S_{A♂} + \omega.R_{A♂}$

(8b) $\dot{T_{A♂}} = -(\mu_{A♂} + \mu^T + \gamma).T_{A♂} + f_3.S_{A♂}$

(8c) $\dot{R_{A♂}} = -(\mu_{A♂} + \omega).R_{A♂} + \gamma.T_{A♂}$

(8d) $\dot{P_{A♂}} = -(\mu_{A♂} + \mu^P).P_{A♂}$

$p_{X♀}$ is the proportion of females in the class X (sub-adults or adults) infected during the first half of gestation. $T_{N,X♀}(t)$ is the number of new cases of transiently infected at time t in females who will be part of the class X (sub-adult or adult) at the time of mating.

(9)
$$p_{X♀}(t) = \frac{\sum\limits_{k=t-170}^{k=t-86} T_{N,X♀}(k)}{\sum\limits_{k=t-170}^{k=t-\frac{1}{\gamma}} T_{N,X♀}(k)}$$

Function of horizontal transmission designated by f_i depends on the sex and age of animals. Thus, according to the contact matrix above, it is considered three forces of infection : f_1 for juvenile females and males, sub-adults females and adults females, f_2 for sub-adult males and f_3 for adults males. N is the total population, T and P denote the set of transiently infected and persistently infected. A frequency-dependent function is used because it is considered that the contact structure is heterogeneous.

(10a) $$f_1 = \left(\beta_T . \frac{T - \tau(t).T_{A♂}}{N - \tau(t).A_{♂}} + \beta_P . \frac{P - \tau(t).P_{A♂}}{N - \tau(t).A_{♂}} \right)$$

(10b) $$f_2 = \left(\beta_T . \frac{T}{N} + \beta_P . \frac{P}{N} \right)$$

(10c) $$f_3 = \left(\beta_T . \frac{T - \tau(t).(T_B + T_Y + T_{Sa♀} + T_{A♀})}{N - \tau(t).(B + Y + Sa_{♀} + A_{♀})} + \beta_P . \frac{P - \tau(t)(P_B + P_Y + P_{Sa♀} + P_{A♀})}{N - \tau(t).(B + Y + Sa_{♀} + A_{♀})} \right)$$

Density dependence

Sigmoig function use for density dependence on the mortality rate of juveniles and the fertility rate of females subadults. I the case of fertility rate, the considered population size is at birth, i.e. two years before.

$$\mu_{Juv}(t) = \mu_{Juv}^{min} + \frac{\mu_{Juv}^{max} - \mu_{Juv}^{min}}{1 + exp(K \times d - d \times N(t))}$$

$$\eta_{Sa}(t) = \frac{\eta_{Sa}^{max}}{1 + exp(d \times N(t - 2yrs) - K \times d)}$$

$$\eta_{SaP}(t) = \frac{\eta_{SaP}^{max}}{1 + exp(d \times N(t - 2yrs) - K \times d)}$$
