# Supplementary material for: A novel epidemiological model to better understand and predict the observed seasonal spread of Pestivirus in Pyrenean chamois populations
Source: Vet Res. 2015 Jul 24;46(1):86. doi: 10.1186/s13567-015-0218-8 (PMC4513621; doi:10.1186/s13567-015-0218-8)
Supplement: Additional file 4: — Data used for estimation by ABC. Tables containing the data used for the parameter estimations by the ABC method. [file 13567_2015_218_MOESM4_ESM.pdf]

Additional file 4 - Data used for estimation by ABC

Numbers of animals by age classes and sex, available in the epidemiological survey for the last eleven years, during spring (Spr.) and autumn (Aut.) (J : juveniles, Sa : subadults, A : adults). Only animals tested for antibodies and viral antigen are take into account for the parameters estimation.

	2000		2001		2002		2003		2004		2005		2006		2007		2008		2009		2010	
	Spr.	Aut.	Spr.	Aut.	Spr.	Aut.	Spr.	Aut.	Spr.	Aut.	Spr.	Aut.	Spr.	Aut.	Spr.	Aut.	Spr.	Aut.	Spr.	Aut.	Spr.	Aut.
J ♂	0	3	0	2	0	3	0	4	0	2	0	3	0	0	0	1	0	0	0	0	0	0
J ♀	0	1	3	2	2	3	1	5	0	2	0	0	0	0	2	2	1	0	0	0	1	0
Sa ♂	0	6	1	0	2	0	0	3	0	7	0	5	0	0	1	9	4	0	0	0	2	0
Sa ♀	0	2	0	2	0	2	0	3	0	3	0	0	0	0	0	1	0	0	0	0	0	0
A ♂	1	2	2	3	1	3	3	4	0	2	0	1	0	0	1	0	0	0	0	0	1	0
A ♀	12	2	6	1	11	8	10	2	13	5	0	4	0	0	5	10	9	0	0	0	10	0
Total	13	16	12	10	16	19	14	21	13	21	0	13	0	0	9	23	14	0	0	0	14	0

Number of animals according to their serological status, aggregated by age classes and sampling seasons. Only data about "antibody +" and "antibody -" were used for the estimation.

	Juveniles		Subadults		Adults	
	Spring	Autumn	Spring	Autumn	Spring	Autumn
antibody +	0	3	2	12	71	45
antibody -	0	21	17	13	15	11
