# Supplementary material for: A novel epidemiological model to better understand and predict the observed seasonal spread of Pestivirus in Pyrenean chamois populations
Source: Vet Res. 2015 Jul 24;46(1):86. doi: 10.1186/s13567-015-0218-8 (PMC4513621; doi:10.1186/s13567-015-0218-8)
Supplement: Additional file 5: — Parameters estimation by ABC. Figure showing the distributions of distances between simulated and observed data. [file 13567_2015_218_MOESM5_ESM.pdf]

Additional file 5 - Parameters estimation by ABC

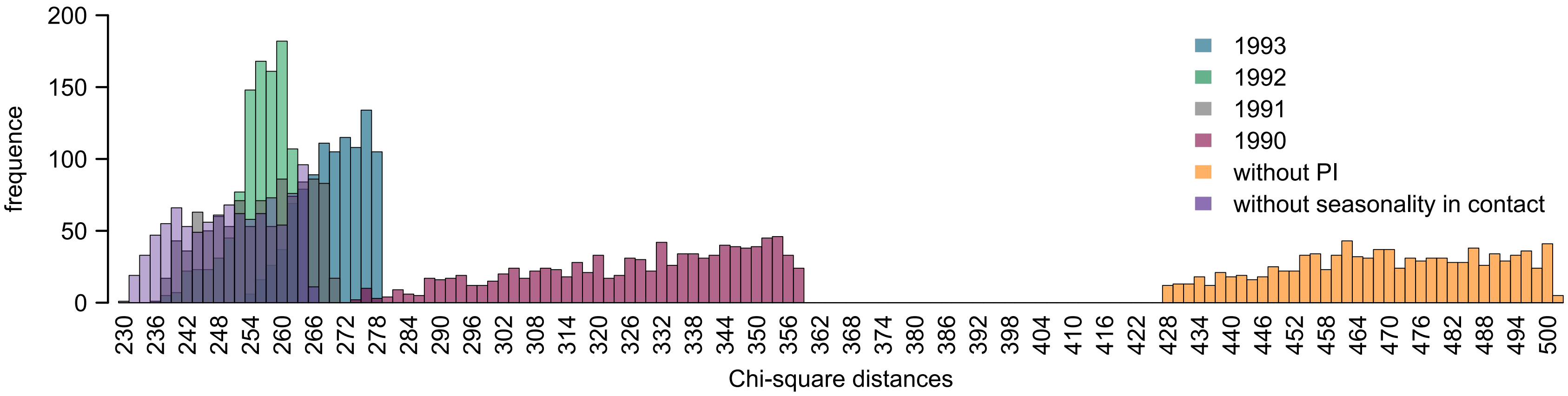

Distributions of distances between simulated and observed data, using the 1% rejection threshold, depending on the year of virus introduction (red : 1990, grey : 1991, green : 1992, blue : 1993, in yellow simulation without the existence of persistently infected animal with an introduction of the virus in 1991, and in purple simulation with homogeneous contacts and an introduction of the virus in 1991).
